# Histone Deacetylation Inhibitors as Therapy Concept in Sepsis

**DOI:** 10.3390/ijms20020346

**Published:** 2019-01-16

**Authors:** Andreas von Knethen, Bernhard Brüne

**Affiliations:** 1 Institute of Biochemistry I, Faculty of Medicine, Goethe-University Frankfurt/Main, 60590 Frankfurt, Germany; b.bruene@biochem.uni-frankfurt.de; 2 Fraunhofer–IME, Project Group Translational Medicine and Pharmacology (TMP), 60596 Frankfurt, Germany

**Keywords:** HDAC, sepsis, epigenetic

## Abstract

Sepsis is characterized by dysregulated gene expression, provoking a hyper-inflammatory response occurring in parallel to a hypo-inflammatory reaction. This is often associated with multi-organ failure, leading to the patient’s death. Therefore, reprogramming of these pro- and anti-inflammatory, as well as immune-response genes which are involved in acute systemic inflammation, is a therapy approach to prevent organ failure and to improve sepsis outcomes. Considering epigenetic, i.e., reversible, modifications of chromatin, not altering the DNA sequence as one tool to adapt the expression profile, inhibition of factors mediating these changes is important. Acetylation of histones by histone acetyltransferases (HATs) and initiating an open-chromatin structure leading to its active transcription is counteracted by histone deacetylases (HDACs). Histone deacetylation triggers a compact nucleosome structure preventing active transcription. Hence, inhibiting the activity of HDACs by specific inhibitors can be used to restore the expression profile of the cells. It can be assumed that HDAC inhibitors will reduce the expression of pro-, as well as anti-inflammatory mediators, which blocks sepsis progression. However, decreased cytokine expression might also be unfavorable, because it can be associated with decreased bacterial clearance.

## 1. Introduction

Sepsis is a major cause of patients’ deaths in intensive care units (ICUs) [1]. It is characterized by organ failure caused by severe infection. One reason for sepsis to occur is a compromised immune system which cannot adequately combat infectious pathogens [2,3]. Sepsis is known as a biphasic disease, first characterized by a hyper-inflammatory phase where high levels of pro-inflammatory cytokines provoke an excessive inflammatory response [4,5]. To limit inflammatory events, a second, hypo-inflammatory phase associated with an immunosuppressive phenomenon follows [6,7]. In septic patients, these two phases can occur in parallel, with a pro-inflammatory predominance at the beginning, changing to an anti-inflammatory prevalence at later time-points [8]. The anti-inflammatory stage is accompanied by T-cell depletion, contributing to immune paralysis [7,9]. This reduced immune status is often reflected by the patients’ predisposition to secondary infections, commonly accompanied by rehospitalizations [10]. Therefore, understanding the mechanisms leading to this immunosuppressed state is mandatory. An impaired immune response as one sequelae of previous sepsis is believed to be a major contributing factor in delayed patients’ deaths [11].

**Figure 1:**
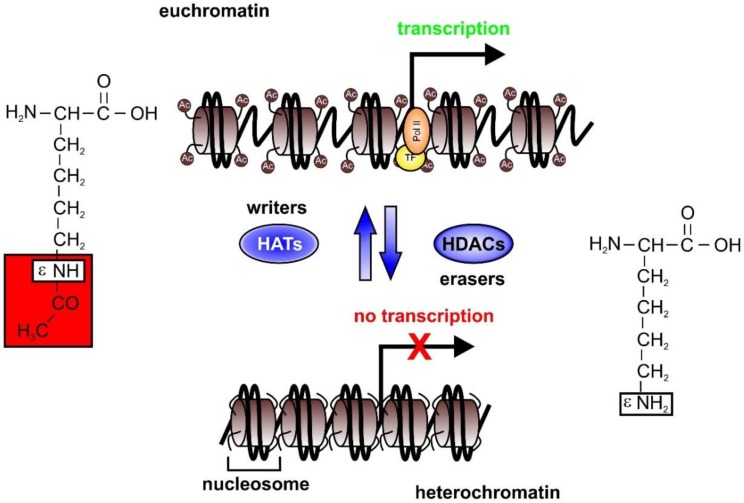
DNA organized in the nucleus, bound to histones, resulting in the formation of nucleosomes. These are closely packed, leading to a transcriptionally inactive state, the hetero-chromatin. Following acetylation of amino-ε lysine residues of histones by HATs—the writers—the nucleosome structure is loosened, which enables transcription factors and the RNA polymerase II to bind to the DNA, which thus initiates transcription. HDACs, also recognized as erasers, can deacetylase lysine-residues of histones, thus counteracting HAT activity and provoking a denser chromatin structure not allowing transcription. (Ac, acetylated; HATs, histone acetyl transferases; HDACs, histone deacetylases; K, lysine; Pol II, RNA polymerase II; TF, transcription factor).

Considering transcriptional regulation of gene expression as the main factor controlling the pro- and anti-inflammatory phenotype of immune cells, it is obvious that altering underlying mechanisms may affect septic outcomes. One prerequisite of transcription is an open chromatin structure designated as “euchromatin” [12]. This allows the recruitment of transcription factors and RNA polymerases to bind to the chromosomal DNA to initiate its transcription (Figure 1). In condensed chromatin, referred to as “heterochromatin”, transcription-factor binding, as well as association of the transcription initiation machinery, is not possible. Upon cell activation, histones located in nucleo- somes of inactive chromatin can be modified by acetylation. This post-translational modification of the ε-amino (ε-NH_2_) group of the lysine is mediated by histone/lysine acetyltransferases (HATs). Due to this modification, the positive charge of lysine is changed, and binding to negatively charged DNA weakened. The acetyl group is transferred from an acetyl-CoA, synthesized by the ATP-citrate lyase (ACLY) [13]. HATs such as CBP/p300, MOF, HBO1, or KAT6A mainly acetylate histones 3 (H3K9ac, H3K14ac, H3K18ac, H3K23ac, H3K27ac, H3K56ac) and 4 (H4K5ac, H4K8ac, H4K12ac, H4K16ac) [14]. Due to this fact, HATs are generally recognized as co-activators of transcription. Acetylation of lysine residues is highly reversible and can be removed by histone deacetylases (HDACs) [15]. This family of 18 enzymes is divided into four groups, based on sequence similarity. HDAC1, 2, 3, and 8 form class I, HDAC4, 5, 6, and 7 constitute class IIa, and class IIb consists of HDAC6 and 10. HDAC11 is the only member of class IV. Finally, class III includes the sirtuins SIRT1–7; whereas the 11 classical HDACs show a Zn^2+^-dependency, the 7 sirtuins are NAD^+^-binding enzymes. To allow deacetylation of histones localized in nucleosomes, HDACs must be in the nucleus. HDAC1,2, and 8 can mainly be found nuclear. In contrast, HDAC3, which is also a member of class I HDACs, is also found in the cytosol. This is also true for all components of class IIa, HDACs 4, 5, 7, and 9, which shuttle between the cytosol and nucleus. Their binding partners are proteins of the 14-3-3 family. Association to 14-3-3 proteins sequesters these HDACs in the cytosol, consequently inhibiting their deacetylating function. HDAC6 and 10, belonging to class IIb, are mainly localized in the cytoplasm. HDAC11 has some sequence similarities with HDACs of class I and II, and thus can be found in the cytosol as well as in the nucleus. A special role of the function of sirtuins has already been suggested because of the special co-factor NAD^+^ from the Krebs cycle in mitochondria, which links the class III HDACs to metabolism. Sirtuins localize to the cytosol, nucleus, as well as to mitochondria. Based on these differences in intracellular localization, HDACs have diverse target proteins, which consequently are not exclusively histones. Here, we focus on the HDACs which deacetylate histones, which belong mainly to the classical family. Characteristics of all four classes of HDACs are summarized in Table 1.

Although HATs and HDACs modify histones, resulting in changing chromatin, i.e., nucleosome structure, this itself is no epigenetic regulation [27]. Epigenetics requires a kind of memory, which is heritable, self-perpetuating, and reversible, and does not alter the DNA sequence [28]. Based on this prerequisite, epigenetic alterations should persist over a longer period. In line with this, the maintenance of the epigenome has been shown to overcome DNA replication and cell division [29].

It is worth mentioning that histone modifications, e.g., acetylations, do not only loosen the chromatin structure of the DNA, but additionally provide a new binding motif for factors such as protein modification readers. One such family of readers are the bromodomain (BRD) and extraterminal domain (BET) proteins, which specifically recognize and bind to acetylated lysine residues on histones [30]. These proteins detect histone acetylations in chromatin, bind to it, and recruit co-factors, transcription factors, and RNA polymerase II to the DNA to modulate gene expression. Besides the BET readers, HATs such as CBP and p300 hold a bromodomain. This means HATs can bind to already acetylated lysine residues, which further enhances their acetylase activity toward histone lysines and allows the recruitment of co-factors as well. Finally, transcription is initiated.

## 2. Epigenetics in Sepsis

### 2.1. HAT and HDAC Activities in Sepsis

**Figure 2:**
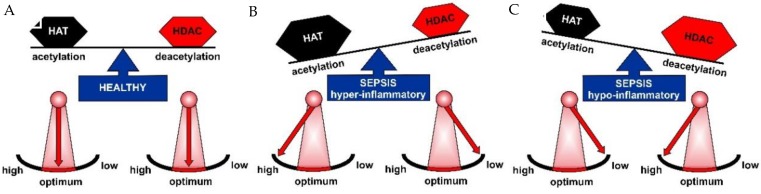
Based on an initial hyper-inflammatory phase, followed by a hypo-inflammatory response which then in part occurs in parallel, epigenetic regulation of gene expression is expected. Thus, compared to the healthy situation (**A**), epigenetics will be out of control due to increased HAT activity in the hyper-inflammatory phase (**B**), enhancing expression of pro-inflammatory genes, and a rise of HDAC-dependent deacetylations (**C**), silencing pro-inflammatory gene expression.

Taking the tremendous changes in gene expression during sepsis initiation and progression into consideration [31,32,33], it is obvious that epigenetic changes contribute to the gene expression profile found in septic patients. Here, it is interesting to differentiate between gene silencing and gene activation mechanisms. The latter one can be triggered by HATs, and the former one is initiated by HDACs. As shown by Warford et al. in autopsies of the brains of sepsis patients, expression of HDAC6 was enhanced [34]. As depicted in Figure 2 in the healthy situation, HAT and HDAC activities are well-balanced. This pattern is changed when, at the beginning of sepsis, an overwhelming expression of pro-inflammatory mediators requires HAT activity to open the chromatin structure for effective transcription of pro-inflammatory genes, such as TNFα, IL-1β, or iNOS [35]. Strikingly, this process is counteracted by the HDACs, which are in part induced and activated by bacterial compounds [36], leading to chromatin reconstitution closely connected to gene silencing [37], which is consequently associated with immunosuppression [38,39].

### 2.2. Polymicrobial Sepsis Mouse Models to Elucidate Epigenetic Mechanisms

Taking a closer look at mechanisms involved in gene activation and gene silencing, mouse models especially have been used. In response to the polymicrobial sepsis model initiated by cecal ligation and puncture (CLP), it was shown to be associated with HDAC6 activation. In line with this, HDACi improved sepsis progression [40,41,42]. Mechanistically, HDAC6, mainly located in the cytosol, has been shown to associate during sepsis with HDAC11 in the nucleus in antigen-presenting cells, inducing IL-10 expression [43,44]. In the control situation, HDAC11 prevents IL-10 expression. Accordingly, the pan-HDAC inhibitor LAQ824, a hydroxamic acid analogue, induces several chromatin changes in macrophages, which leads to enhanced HDAC11 recruitment to the IL-10 promoter in Balb/c mice [45]. Also, other HDACis belonging to the hydroxyamic acid family of compounds, which all are pan-HDACis, such as panobinostat and TsA, inhibited IL-10 production in peritoneal elicited macrophages (PEM) following LPS stimulation [45]. The more specific HDACi MS-275, inhibiting class I HDACs, did not effectively prevent IL-10 expression. In parallel, LAQ824 enhanced LPS-mediated expression of pro-inflammatory cytokines, such as TNFα, IL-6, IL-1α/β, and RANTES [45]. Considering these alterations, it is obvious that HDACis shapes the expression profile of macrophages to a pro-inflammatory expression pattern. Interestingly, as a possible consequence of this shift, LAQ824-treated PEM effectively prime naïve antigen-specific T-cells. Moreover, anergic T-cells recover responsiveness. Considering the biphasic nature of sepsis, i.e., a hyper-inflammatory vs. a hypo-inflammatory response, the latter one, especially, might be effectively improved by HDACis.

One characteristic of hypo-inflammation is immune-paralysis, mainly mediated by T-cell depletion. This leads to an inappropriate immune response toward the initial, or a new second infection [46,47]. Therefore, recovery of T-cell function by inhibiting T-cell apoptosis and preventing anergy will improve septic outcomes [9,48]. Although HDACis have already been clinically approved, this therapy approach focuses only on tumor treatment [47,49] and a spectrum of other diseases [50]. Up to now, there have been no clinical trials listed using HDACis to treat sepsis. However, HDACis have already been used concerning their effect on parasite growth, such as Plasmodium, Leishmania, and Schistosoma [51], as well as to prevent human immunodeficiency virus (HIV) latency [52,53]. In general, epigenetic manipulations are considered to have therapeutic potential in infectious diseases [54]. Most importantly, the correct moment in sepsis onset and progression to inhibit HDACs has to be found. As shown in Figure 2A, the balance between HDACs and HATs is important to guarantee an appropriate immune response. Any alteration leading to a predominance of either HATs (Figure 2B) or HDACs (Figure 2C) is associated with corresponding epigenetic modifications, such as gene activation or gene silencing. In the sepsis situation, gene activation is mainly valid in the hyper-inflammatory phase, whereas gene silencing occurs particularly during immune paralysis in the hypo-inflammatory response. Taking this together, it is obvious that epigenetic regulation is an important mechanism during sepsis progression.

### 2.3. Endotoxemia and LPS Treatment of Cells to Mimic Epigenetic Alterations in Sepsis

Besides polymicrobial sepsis models, such as CLP, colon ascendens stent peritonitis (CASP), or peritoneal cavity infection (PCI) [55], endotoxemia by a LPS challenge is an important model mimicking sepsis-like symptoms in animals. LPS treatment is a more controllable model, which can be used to understand underlying principles leading to sepsis-dependent cellular modifications [56].

Besides animal models that are also cellular in vitro models, focusing on the role of macrophages is used to understand the role of HATs, HDACs, and HDACis in sepsis. In bone-marrow-derived macrophages, Aung et al. found that LPS regulates pro-inflammatory gene expression in macrophages by altering histone deacetylase expression [57]. In this study, the authors observed that LPS transiently repressed expression of HDACs 4, 5, and 7, followed by an induction of these HDACS, which was more rapid, concerning HDAC-1 mRNA [57]. Recently, Wu et al. described the crucial role of HDAC2 in LPS-dependent inflammatory activation of macrophages [58]. The expression of HDAC2, belonging to class I of HDACs, is enhanced following macrophage stimulation with LPS. Knockdown of HDAC2 reduces expression of pro-inflammatory genes IL-12, TNF-α, and iNOS [58]. This is in line with the work of Somanath et al. [59,60], showing a similar effect after CRISPR/Cas9-mediated HDAC2-disruption. Moreover, adoptive transfer of macrophages with a HDAC2 knockdown to mice diminishes their inflammatory response to LPS and *E. coli* [58]. Mechanistically, HDAC2 reduced c-Jun expression by directly binding to its promoter. There, acetylation of histones is removed, leading to compact nucleosome formation and, consequently, to gene-silencing following LPS-treatment. Considering LPS tolerance or cellular reprogramming as a mechanism associated with endotoxemia, it is interesting that the gene expression signature characteristic for endotoxin tolerance was also found in patients during the early onset of sepsis [61]. This is especially important, because endotoxin tolerance has been assumed to be mediated in part by epigenetic alterations, also termed “trained immunity” [62,63]. HDAC3 has been found to be required for the inflammatory gene expression program in macrophages [64]. In macrophages which do not express a functional HDAC3, roughly 50% of the pro-inflammatory genes in response to LPS were not expressed [64]. Interestingly, this was mediated in a large part by the loss of basal and LPS-dependent expression of IFNβ, suggesting the involvement of STAT1 as a contributing transcription factor. Also, HDAC7 seems to be involved in TLR4-dependent pro-inflammatory gene expression. As shown by Shakespear et al., HDAC7 promotes pro-inflammatory gene expression in mouse macrophages following LPS treatment [65]. HDAC7 was elevated in PEMs compared to untreated BMDMs. Mechanistically, HDAC7 seems to link LPS signaling with HIF-dependent transactivation [65].

One further mechanism of LPS-dependent epigenetic alterations is reactive oxygen species (ROS)-mediated activation of HDAC3, leading to TNF-α expression in cardiomyocytes [66]. ROS released from mitochondria activate c-Src signaling, finally activating HDAC3 [66].

### 2.4. Glucocorticoids as Epigenetic Regulators in Sepsis

Considering sepsis as a mainly catabolic condition, Alamdari et al. observed that, during sepsis in rats, expression and activity of HDAC 6 was downregulated in skeletal muscle, whereas HAT p300 expression was upregulated [35]. Mechanistically, the glucocorticoid receptor antagonist RU38486 reversed this expression change. In line with this, treatment of the rats with dexamethasone significantly enhanced the expression of p300 and reduced expression of HDAC6 [35]. For further analogy, Yang et al. (2007) demonstrated that proteolysis of cultured myotubes was induced by dexamethasone [67]. In cultured L6 myotubes, dexamethasone induced increased nuclear localization of p300 and downregulated expression of HDAC3 and 6.

### 2.5. Role of Sirtuins in Sepsis

Sirtuins, i.e., class III HDACs, are largely uninvolved in histone deacetylation. Thus, other different roles have been defined. Among these other roles, HMGB1 hyperacetylation has been attributed to the function of SIRT1. This is a prerequisite for HMGB1 release from the cells. This process is also triggered by LPS stimulation, and is also valid in an animal model of polymicrobial sepsis [68]. Analogous to this work, Zhao et al. provided evidence that SIRT1-specific inhibition by EX-527 significantly improved survival of mice following CLP [69]. Moreover, expression of pro-inflammatory cytokines TNF-α and IL-6 in the blood and peritoneal fluid were reduced [69]. Interestingly, sepsis-dependent coagulopathy, as well as bone marrow atrophy, were reduced [69]. More obviously, a role of SIRTs has been proposed in immune-metabolism [70] or by long-noncoding RNA [71]. Interestingly, SIRT2 deficiency prevents chronic staphylococcus infection [72]. It has also been shown that acute kidney injury in a septic rat model is in part due to the reduced activation of SIRT1 and 3, giving rise to enhanced acetylated SOD2 levels, concomitant oxidative stress, and mitochondrial damage [73]. The chemical SIRT1 activator, resveratrol, restored SIRT1/3 activity and improved rat survival [73]. These data support the notion that members of the sirtuin family of HDACs mainly deacetylate proteins others than histones.

In summary, the regulation of gene expression during sepsis requires the balanced function of HATs and HDACs [38,39]. An overshooting of both sides is deleterious, associated with a bad septic outcome. Taking this into consideration, altering the function of HDACs may be one new tool to restore appropriate gene expression and to maintain a functional adequate immune response.

## 3. HDAC Inhibitors (HDACi) as Anti-Inflammatory Agents

Taking the role of epigenetic modifications during sepsis initiation and progression into consideration, it is obvious that HDAC inhibitors (HDACi) will be effective in altering pro- and anti-inflammatory gene expression. Considering the broad range of unspecific, so-called “pan” HDAC inhibitors, and some more recently developed specific ones (as shown in Table 2), the role of HDAC inhibition could be determined. Initial studies have used the pan-HDAC inhibitors, SAHA (vorinostat) and trichostatin A (TSA) in various models of sepsis, as summarized in Table 2; these three compounds belong to two different chemical classes of HDAC inhibitors. Both compounds were effective in improving sepsis outcomes. Following CLP operation, the survival was improved in response to SAHA [74]. SAHA reduced TNF-α and IL-6 expression in LPS-endotoxemia [75]. Neuronal damage was also reduced by SAHA treatment of CLP-operated animals [76]. A similar protective role was shown with the HDACi TSA [76]. In LPS-dependent endotoxemia, acute lung injury and inflammation were reduced after the application of TSA [77]. This protective role was also evident in bone-marrow-derived macrophages (BMDM) by blocking DNA fragmentation, and reduced expression of pro-apoptotic genes [77]. In this cell type, TSA enhances LPS-dependent Cox-2, Cxcl2, and Ifit2 expression, whereas it blocks the expression of the LPS target genes, Ccl2, Ccl7, and Edn1 [57]. In the CLP model, TSA improved survival, reduced acute lung injury, and lowered the expression of TNF-α and IL-6. Moreover, expression of TLR2, TLR4, and the adaptor protein MyD88 were attenuated. Concomitantly, nuclear NF-κB was reduced [78]. In another study, the authors observed reduced plasma urea and creatinine, a decrease of CRP, less tubular damage, and reduced expression of MCP-1 and HDACs2/5. In line with this, H3Ac was enhanced [79]. Moreover, TSA reduced neutrophil infiltration, ICAM-1, and E-selectin expression [80], and reduced liver-damage markers, IL-10 expression, and MPO [81]. Interestingly, TSA blocks endotoxin tolerance induction as well [82]. Other pan-HDAC inhibitors, such as valproic acid [83] or butyric acid [80,84] were similarly effective in improving septic outcomes by reducing pro-inflammatory gene expression and concomitant reduced organ damage. However, unwanted side effects, such as enhanced toxicity, prevent their use in clinical trials [85].

The use of HDACi, which are specific for one class of HDACs or only one HDAC directly, is gaining more interest [86]. As seen in Table 2, currently, the HDAC6 inhibitor tubastatin A is particularly important. Likewise, showing a similar protective role, such as the pan-HDACi SAHA and TSA, only HDAC6 is inhibited [87,88,89].

Although SIRTs are barely involved in histone deacetylation, their specific inhibition improved the septic outcome in rodents as well. CLP-mediated damage was restored by the SIRT1-specific inhibitor EX-527 [90], and the SIRT2-specific inhibitor AGK2 [91]. With the use of more specific HDACi, there should be a more precise target which is affected. Side effects should therefore be minimized. However, up to now, there have been no clinical trials using HDACi to treat sepsis.

## 4. Conclusions

A therapy approach to fine-tuning gene expression by the activating or silencing of genes is a promising tool to overcome dysregulated gene expression, as observed in sepsis. Because accurate tuning of gene expression is mandatory, the development of new, more specific HDAC inhibitors is important. This will allow a direct and reversible change in gene expression, which is necessary to prevent sepsis progression and improve sepsis outcomes. Therefore, the use of HDAC inhibitors in clinical trials will be one major method in the near future for clarifying the impact of epigenetics during sepsis initiation and progression.

## Figures and Tables

**Table 1:** HDAC classification, based on sequence similarities.

	Superfamily	Family	Class	Subclass	Protein	Ref.
CLASSICAL	Arginase/ deacetylase superfamily	Histone deacetylases	Class I		HDAC1, 2, 3, 8	[16,17,18,19]
			Class II	a	HDAC4, 5, 7, 9	[20,21,22]
				b	HDAC6, 10	[21,23]
			Class IV		HDAC11	[24]
	Deoxyhypusine synthase like NAD^+^-binding domain superfamily	Sir2 regulators	Class III	I II III IV	SIRT1, 2, 3 SIRT4 SIRT5 SIRT6, 7	[25] [25] [25] [26]

**Table 2:** HDAC inhibitors (HDACi) used to treat sepsis (↓: decrease; ↑: increase).

	HDACi	Inhibition	Model	HDACi Effect	Ref.
Hydroxamic acids	SAHA (Vorinostat)	pan	CLP	survival↑	[74]
			LPS-endotoxemia	TNF-α↓, IL-6↓	[75]
			Long-term survival following CLP	Long-term survival↑	[75]
			CLP/SAE	neuronal apoptosis↓; locomotive activity↑, H3Ac, H4Ac; nuclear HDAC4↑; Bax↓, Bcl-XL↑	[76]
	TSA	pan	LPS endotoxemia	ALI↓, apoptosis↓, inflammation↓	[77]
			LPS/BMDM	DNA fragmentation↓, expression of apoptotic/pyroptotic genes↓	[77]
			CLP	survival↑, ALI↓, TNF-α↓, IL-6↓, TLR2↓, TLR4↓, MyD88↓, nuclear NF-κB↓, I-κBα↓	[78]
			Tolerance (LPS/THP-1 cells)	IL-6↑, IL-10↓	[82]
			LPS-induced ALI	IL-1β↓, TNF-α↓, lung MPO↓, PMN cells in BALF↓	[92]
			LPS-induced ALI	inflammation↓, ALI↓, survival↑	[93,94]
			CLP/SAE	neuronal apoptosis↓, locomotive activity↑, H3Ac↑, H4Ac↑, nuclear HDAC4↑; Bax↓, Bcl-XL↑	[76]
			CLP	plasma urea↓, creatinine↓, CRP↓, tubular damage↓, TNF-α↓, MCP-1↓, BMP-7↑, HDAC2/5↓, H3Ac↑	[79]
			CLP	ALI↓, neutrophil infiltra-tion↓, ICAM-1↓, E-selec-tin↓, IL-6 ↓, survival↑	[80]
			CLP	ALT/AST↓, MDA↓, MPO↓, ICAM-1↓, IL-6↓, IL-10↓	[81]
			LPS/BMDM	Cox-2↑, Cxcl2↑, Ifit2↑, Ccl2↓, Ccl7↓, Edn1↓	[57]
Benzamides	TubA	HDAC6	CLP	circulating monocytes↑, lymphocytes↑, granulo-cytes↓	[89]
			“two-hit” model	survival ↑, MPO↓, TNF-α ↓, IL-6 ↓	[40]
			CLP	survival↑, ALI↓ MPO↓, TNF-α↓, IL-6↓, MΦ apoptosis↓, bacterial clearance↑, splenocyte phagocytosis↑	[41]
			CLP	innate immune cells↑, MΦ↑, neutrophils↑	[88]
			Long-term survival following CLP	B cells↑, innate immune cells↑, MΦ↑	[87]
	MS-275 (entinostat)	HDAC1,2,3	CLP	not improved	[41]
			LPS-dependent AKI		[95]
	KBH-A42	pan	LPS-endotoxemia	TNF-α↓, IL-1β↓, IL-6↓, iNOS↓	[96]
Cyclic peptides	Romidepsin	pan	comorbidity sepsis		[97]
Short chain fatty acids	Valproic acid	pan	LPS-dependent AKI	histological scores↓, MPO↓, NF-κB p65↓, NO↓, iNOS↓, TNF-α↓, IL-1β↓, nuclear HDAC3↓, cytosolic HDAC3↑	[83]
	Butyric acid	pan	CLP	ALI↓, neutrophil infiltra-tion↓, ICAM-1↓, E-selec- tin↓, IL-6 ↓, survival↑	[80]
			CLP	long-term cognitive impairment↓	[84]
SIRT-specific	EX-527	SIRT1	CLP	survival↑, TNF-α↓, IL-6↓, coagulopathy↓, bone marrow atrophy↓	[69]
	AGK2	SIRT2	CLP	survival↑, TNF-α↓, IL-6↓, clot formation↓, platelet function↓, bone marrow atrophy↓	[91]

## Abbreviations

Ac	acetylation
AKI	acute kidney injury
ALI	acute lung injury
ALT	alanine amino transferase
AST	aspartate amino transferase
BALF	bronchoalveolar lavage fluid
BET	bromodomain and extraterminal domain
BRD	bromodomain
CBP	CCAAT-binding protein
CLP	cecal ligation and puncure
CRP	C reactive protein
DC	dendritic cell
H	histone
HAT	histone acetyltransferase
HBO	histone acetyltransferase binding to ORC1
HDAC	histone deacetylase
ICU	intensive care unit
K	lysine
KAT6A	lysine acetyl transferase 6A
LPS	lipopolysaccharide
MΦ	macrophage
MDA	malondialdehyde
MOF	males absent on the first
MPO	myeloperoxidase
NAD	nicotinamide adenine dinucleotide
SAHA	suberoylanilide hydroxamic acid
SAE	sepsis associated encephalopathy
SIRT	silent information regulator
TLR	toll-like receptor
TSA	TrichostatinA
TubA	Tubastatin A
